# Docosahexaenoic Acid, a Potential Treatment for Sarcopenia, Modulates the Ubiquitin–Proteasome and the Autophagy–Lysosome Systems

**DOI:** 10.3390/nu12092597

**Published:** 2020-08-26

**Authors:** Jung Hoon Lee, Jun Hyoung Jeon, Min Jae Lee

**Affiliations:** 1 Department of Biochemistry and Molecular Biology, Seoul National University College of Medicine, Seoul 03080, Korea; jjh_1995@snu.ac.kr; 2 Neuroscience Research Institute, Seoul National University College of Medicine, Seoul 03080, Korea; 3 Department of Biomedical Sciences, Seoul National University Graduate School, Seoul 03080, Korea

**Keywords:** sarcopenia, docosahexaenoic acid (DHA), omega-3 polyunsaturated fatty acid (PUFA), proteolysis, proteasome, autophagy, ubiquitin, proteostasis

## Abstract

One of the characteristic features of aging is the progressive loss of muscle mass, a nosological syndrome called sarcopenia. It is also a pathologic risk factor for many clinically adverse outcomes in older adults. Therefore, delaying the loss of muscle mass, through either boosting muscle protein synthesis or slowing down muscle protein degradation using nutritional supplements could be a compelling strategy to address the needs of the world’s aging population. Here, we review the recently identified properties of docosahexaenoic acid (DHA). It was shown to delay muscle wasting by stimulating intermediate oxidative stress and inhibiting proteasomal degradation of muscle proteins. Both the ubiquitin–proteasome and the autophagy–lysosome systems are modulated by DHA. Collectively, growing evidence indicates that DHA is a potent pharmacological agent that could improve muscle homeostasis. Better understanding of cellular proteolytic systems associated with sarcopenia will allow us to identify novel therapeutic interventions, such as omega-3 polyunsaturated fatty acids, to treat this disease.

## 1. Introduction

During the aging process, while slow-twitch muscle fibers (type I, which relies on aerobic respiration for muscle endurance) remain largely unchanged, the mass of fast-twitch fibers (type II, which determines muscle power) is significantly reduced via the progressive denervation and reinnervation processes [1]. In addition, the complex fiber-type transformation, which provides plasticity to muscles to adapt to developmental and environmental changes, requires tightly regulated proteolysis to remove the existing fibers, and this pathologic acceleration of proteolysis is implicated in sarcopenia, the age-related loss of muscle mass and function [2,3]. In sarcopenic muscle, type II fibers decrease much faster than type I. In 2019, European Working Group of Sarcopenia in Older People (EWGSOP2) classified sarcopenia into three stages: probable sarcopenia, confirmed sarcopenia, and severe sarcopenia [4]. However, despite its intuitive nosological definition, a consensus on the operational definition of sarcopenia has yet to be achieved.

Based on the meta-analysis, the overall prevalence of sarcopenia is approximately 10% in the population aged 60 years or older (without gender differences) [5]. Given this high prevalence and the fact that ~2.1 billion people are expected to be more than 60 years old by the year 2025 [6], sarcopenia will be a major healthcare issue for both patients and the society. Therefore, in addition to physical exercise, nutritional strategies are uniquely important as an effective preventive measure against sarcopenia, as well as the accompanying frailty and disabilities. The homeostatic imbalance between protein synthesis and degradation in the geriatric muscle probably originates from dysregulation of complex signaling pathways [7,8,9]. Therefore, understanding the mechanisms of sarcopenia is essential to identify the targets for pharmacological interventions to prevent or treat sarcopenia.

The primary treatment approach is resistance exercise. Previously, endurance training was considered not effective to improve muscle mass or strength, but it is now generally accepted that the ATP-producing endurance training and balance training, combining resistance and endurance trainings, are both preventive and therapeutic to age-induced sarcopenia of skeletal muscles [10]. Physical training can restore the aged muscle’s sensitivity to protein intake, which subsequently produces anabolic stimuli to facilitate muscle protein synthesis. Potentially effective substances include anabolic steroids, myostatin (natural muscle growth antagonist) inhibitors, ghrelin agonists, and antioxidants. Several supplements have been suggested to produce beneficial muscle regenerative effects, for example, essential amino acids, such as leucine, creatine monohydrate, omega-3 polyunsaturated fatty acid (PUFA), vitamin D, vitamin B_6_, folic acid, and magnesium [11,12]. De Spiegeleer et al. identified seven systematic reviews or meta-analyses, and found that vitamin D and testosterone can improve muscle mass, muscle strength and physical performance in subjects aged over 65 years [13].

Omega-3 PUFAs have been shown to reduce the development of sarcopenia in the older population by positively modulating intracellular metabolic signals [14,15]. However, how omega-3 PUFAs affect the cellular protein catabolism has not extensively studied yet on the molecular level. We previously reported that docosahexaenoic acid (DHA), a major dietary omega-3 PUFA, effectively delayed proteasomal degradation of muscle proteins in a cellular atrophy model [16]. The inhibitory effect of DHA on protein degradation might originate from the generation of excess proteasome substrates through oxidation, which suppresses cellular proteasome activity by accumulating the hard-to-degrade substrates. On the contrary, DHA appears to induce autophagy in many cancer cell lines via p53-mediated AMPK/mTOR signaling [17]. This phenomenon may reflect the negative feedback communication between the two catabolic systems, which are not independent, but are, in fact, connected by a highly regulated negative feedback crosstalk [18,19,20]. In this article, we review the mechanisms of sarcopenia development and progression in the context of protein homeostasis (proteostasis), focusing on DHA as a novel sarcopenia-targeting molecule. We address the recent understanding of muscle protein degradation via the ubiquitin–proteasome system (UPS) and the autophagy–lysosome system (ALS) during sarcopenia.

## 2. Molecular Mechanisms of Sarcopenia Development and Progression 

### 2.1. General Consideration

The pathogenesis of sarcopenia, one of the leading causes of disability in the older adults, is still not completely understood. Several suggested etiological factors include decreased numbers of satellite cells (adult myogenic stem cells), increased inflammation or cytokines, and abnormality in endocrine function or neuromuscular function [21,22,23,24,25]. Protein-energy malnutrition may also trigger the loss of muscle mass and strength during the aging process [26]. Evidence suggests that genetic variation is another factor that influences the individual and group differences in sarcopenia susceptibility [27]. In addition, the impairment of multiple intracellular (both anabolic and catabolic) signaling pathways appears to be involved in muscle mass loss. Even the relatively healthy seniors lose ~1% of muscle mass and ~3% muscle strength annually [28]. Considering these factors, sarcopenia is similar to other metabolic diseases such as obesity, type II diabetes, and nonalcoholic fatty liver disease [29,30,31,32]. 

While multiple mechanisms certainly contribute to the etiology, the major risk factor of sarcopenia is age. Moreover, muscle loss is one of the first manifestations of tissue or organism aging. The key molecular and cellular properties, underlying the progressive and physiological decline of all living organisms, are a steady-state increase in inflammation, oxidative stress, DNA mutations, dysfunctional mitochondria, senescent cells, and uncontrolled proteostasis [33]. At the molecular level, skeletal muscle mass is directly dependent on the rates of protein synthesis and degradation. Since humans have no protein storage pool and the skeletal muscle serves as the major deposit of proteins, protein metabolism becomes even more critical as an adaptation to environmental changes. Therefore, the onset of sarcopenia is the manifestation of the imbalance between metabolic processes. Intrinsic factors within skeletal muscle (e.g., inflammation, apoptosis, proteasomal and lysosomal function, and calcium metabolism) and extrinsic factors in systemic environments (e.g., cytokine levels, hormonal changes, and diminished nutrient intake) are intricately interconnected to contribute to the progression of sarcopenia [34,35,36,37,38,39]. 

**Figure 1:**
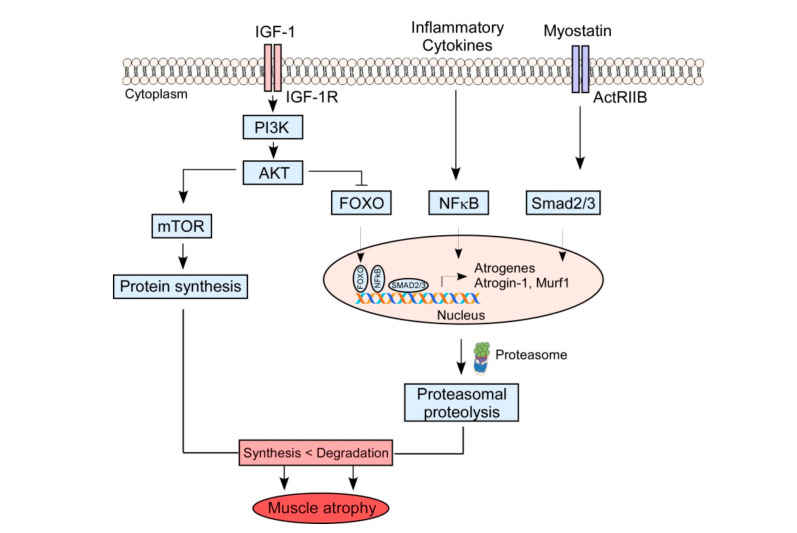
Putative molecular mechanisms involved in the development of sarcopenia during aging. Skeletal muscle protein homeostasis is regulated by balancing protein synthesis and degradation. Insulin-like growth factor I (IGF1)-Akt pathway controls muscle growth via activating the kinase mammalian target of rapamycin (mTOR) and inhibiting the members of the class O of forkhead box transcription factors (FOXO) family. FOXO transcription factors are required for the transcriptional upregulation of atrophy-related genes (atrogenes), ubiquitin ligases atrogin-1, and muscle ring finger 1 (MuRF1), leading to the ubiquitylation of muscle proteins and their degradation by the proteasome. Atrogin-1 and MuRF1 are also induced by several other signaling cascades, including the inflammatory cytokine-NF-κB and the myostatin-SMAD signalings. During aging, protein homeostasis is disrupted, shifting the balance between protein synthesis and protein degradation, leading to increased protein degradation and resulting in muscle atrophy, or sarcopenia.

In healthy subjects, there is a clear correlation between the protein synthesis rate and the levels of growth hormones, such as insulin-like growth factor-I (IGF-I) in skeletal muscle [40,41] (Figure 1). In aged muscle, low levels of IGF-I are often accompanied by a high expression of muscle growth inhibitors, including the pro-inflammatory cytokines tumor necrosis factor α and interleukin 6 [42,43]. IGF-I downregulation is also closely associated with enhanced myostatin levels [44,45,46] and reduced DNA/protein synthesis [47]. The serum or skeletal muscle mRNA levels of myostatin increase with aging and are inversely correlated with the skeletal muscle mass [48,49]. Systemic overexpression of myostatin induces significant muscle loss in adult mice, analogous to human cachexia syndromes [50]. Consistent with myostatin being a myogenesis-inhibiting myokine, aged mice treated with the myostatin-neutralizing antibodies show significantly increased skeletal muscle performance and mass [51,52]. Therefore, pharmacological modulation of, or nutritional intervention with muscle protein synthesis is a promising strategy to restore sarcopenic muscles. 

### 2.2. Muscle Protein Catabolism

A common feature of aging is the progressive loss of cellular proteostasis [53]. It is evident that anabolic sensitivity of muscle protein synthesis to protein intake or physical exercise is gradually reduced during aging [54,55]. A decreased production of circulating and tissue-associated growth hormones, such as IGF-I, may be connected to this phenotype as well [56,57,58]. In addition to the alteration of muscle protein synthesis rate, dysregulation of proteolysis has also been reported during sarcopenia [59,60,61]. Many studies also point that the age-dependent increase of inclusion formation might be directly related to decrease of cellular proteasome and autophagy [62]. The UPS and ALS are two major intracellular proteolytic pathways in eukaryotes. The UPS primarily removes short-lived proteins, such as cell cycle regulators and transcription factors, as well as misfolded proteins from the cytosol, nucleus, and endoplasmic reticulum [63,64]. In contrast, the ALS removes relatively long-lived proteins and protein complexes, protein aggregates, and cellular organelles [20,65]. Both systems are highly conserved eukaryotic protein recycling machineries, where the substrates are degraded inside the proteasomes or the autolysosomes.

Another general feature of aging cells is the accumulation of oxidized proteins in the proteome. Whether this is the result of impaired UPS and ALS during aging or, the opposite, the cause of decreased activity outputs of these catabolic systems, is not clear. When young and aged mice or rats were compared, some studies suggested that proteasome activity in the skeletal muscle appeared to decrease in the geriatric group [66,67,68,69]. However, most of these studies measured proteasome activity based only on 20S, but not 26S proteasomes, and used simple total protein levels to normalize data. Therefore, the observed results might originate from the age-related reduction of overall biological functions, not specifically reflecting proteasomal activity. This also contradicts the observation that muscle protein ubiquitylation and degradation are markedly increased in the mouse model of muscle atrophy [24,70]. 

**Figure 2:**
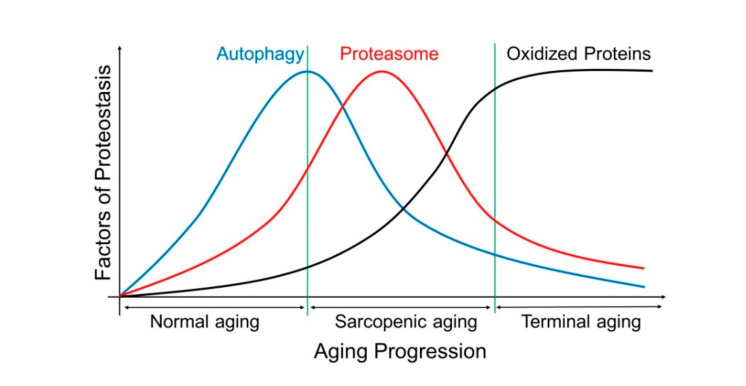
A hypothetical model depicting the changes in proteasome (red) and autophagy (blue) activity, as well as in the level of cellular oxidized proteins (potentially indicating overall cellular oxidative stress; black) in muscle. The thresholds for the onsets of muscle atrophy and sarcopenia are presented as the green vertical lines. In this model, the occurrence of sarcopenia is preceded by accumulation of oxidized proteins and subsequent impairment of the ubiquitin-proteasome system (UPS) and autophagy-lysosome system (ALS). The induction of proteasome activity (as well as autophagic flux) under the physiological conditions possibly reflects the adaptive response to oxidative stress although controversies exist about the changes of proteasome and autophagic flux during aging. The feedback regulatory circuit between the two proteolytic systems and the regulatory mechanism contributing their balance during sarcopenia remains to be determined.

The Goldberg group and others reported the increasing tendency of protein degradation during aging or after denervation, along with higher contents and activities of proteasome in muscle, demonstrating its contribution to the degradation of myofibrillar proteins [71,72,73,74]. There are conflicting reports regarding proteasome activity changes during the aging process [75,76,77,78,79]. We hypothesize that, in muscle-wasting conditions, the proteolytic function, mainly the UPS, appears to be enhanced, similar to other pathological conditions, such as sepsis, cancer, burns, diabetes, and starvation [80], while during the late aging stage, the overall proteolytic activity may be suppressed down to the threshold levels (Figure 2). Consistently, the age-dependent proteasome activity in rats was steadily increased up to 29 months of age and then decreased during the senescent stage [78]. 

### 2.3. The Ubiquitin–Proteasome System (UPS)

**Figure 3:**
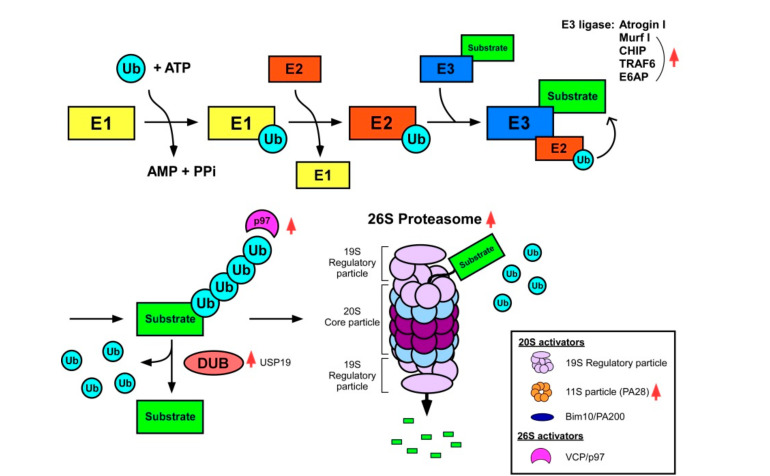
Muscle protein degradation by the UPS. The sequential enzymatic cascade reactions conjugate multiple ubiquitin moieties to the protein substrate. Polyubiquitylated substrates are either processed by deubiquitylating enzymes (DUBs) or degraded by the proteasome. The UPS components, such as ubiquitin ligases and proteasome regulators implicated in muscle atrophy, are shown. The increase in various components in ubiquitin–proteasome system is observed in sarcopenia, contributing to excessive and sustained protein degradation in skeletal muscle, shown in red arrows.

The rate-limiting step of UPS-mediated proteolysis is substrate ubiquitylation by E3 ubiquitin ligases. These ligases mark the target proteins with multiple ubiquitin moieties to be recognized by the proteasome for degradation (Figure 3). Two muscle-specific E3 ubiquitin ligases, the muscle atrophy F-box (MAFbx, also known as atrogin-1, an atrophy agent) and muscle RING Finger 1 (MuRF1), are among the genes regulated during muscle atrophy [81,82]. A significant correlation between their expression and the onset of muscle atrophy were reported (~40-fold increase in atrogen-1 and a ~20-fold increase in MuRF1) [83,84]. Both E3 ubiquitin ligases were transcriptionally upregulated in rodent models, as well as in human patients in response to diverse atrophic stimuli, including myostatin/TGF-β signaling [85]. For example, the loss of muscle mass after muscle denervation was reversed in atrogin-1-null mice [83]. Furthermore, nullification of *MuRF1* in mice attenuated muscle atrophy during aging [67], as well as in several atrophy-induced conditions, such as denervation [83,86], immobilization [87] and glucocorticoid treatment [88]. Atrogin-1 promoted degradation of MyoD, a key muscle differentiation transcription factor, and of eukaryotic initiation factor 3 subunit 5 (eIF3F), an important activator of muscle protein synthesis [89,90]. MuRF1 ubiquitylated a variety of sarcomere proteins such as troponin I, myosin heavy and light chains, actin, and myosin binding proteins [89]. However, deletion of these E3 ubiquitin ligases only led to limited effects on the muscle protein degradation [83], suggesting that a different strategy, for example, targeting the downstream proteolysis, such as proteasomes, is required for more effective treatment of sarcopenia. Considering the antagonistic role of deubiquitylating enzymes (DUBs) on E3 Ub ligases, identification of cognate DUBs for atrogin-1 and MuRF1 and their activation may potentially inhibit UPS-mediated sarcopenic muscle protein degradation. 

Various transcription factors are also involved in the regulation of atrogin-1 and MuRF1mRNA expression, including the transcription factor forkhead box O3 (FOXO3). During muscle wasting conditions, Akt phosphorylates the FOXO transcription factors and consequently prevents their nuclear translocation, resulting in downregulation of atrogin-1 and MuRF1 expression [81]. It is notable that in skeletal muscle, *FOXO3*, one of the most prominent human longevity-associated genes, plays a critical role in the expression of genes involved in the UPS, ALS and ROS stress responses during the catabolic conditions [91,92]. The FOXO transcription factors also upregulate the mitochondrial ubiquitin ligase Mul1, the ligase essential for mitophagy, either during fasting or other catabolic conditions [93].

Although the contributions of atrogin-1, MuRF1, and other E3 ligases, such as TNF receptor-associated factor 6 (TRAF6) [94], the C-terminus of Hsc70 interacting protein (CHIP/STUB1), and E6-associated protein (E6AP) [72], to muscle atrophy have been widely studied, little is known about how 26S proteasome affects muscle protein degradation during aging. Proteasomes are generally regarded as highly abundant and stable [95,96]. The 26S proteasome consists of a barrel-shaped protein complex (the 20S core particle, CP) responsible for proteolysis, and one or two ATPase-containing complexes (the 19S regulatory particle, RP) involved in substrate recognition, unfolding, and translocation. Proteasome inactivation through conditional knockout (KO) of a non-ATPase subunit Rpt3, unexpectedly, induces muscle growth defects and loss of muscle force production, rather than attenuating muscle protein breakdown [97]. This phenomenon is accompanied by upregulation of atrogin-1 and MuRF1 and impaired autophagosome formation, both potentially accounting for skeletal muscle degeneration and muscle weakness in the Rpt3 KO mice. 

Dysregulation of proteasome activators has also been observed in aged muscles. For example, impaired interaction and activation of 20S proteasomes by the proteasome activator 28αβ complex (PA28αβ, also known as 11S) resulted in the age-related muscle atrophy [98]. Valosin-containing protein VCP/p97, an ATPase/unfoldase, is another important regulator of the 26S proteasome-mediated muscle protein degradation and is involved in escorting substrates to the 26S proteasome by extracting ubiquitylated proteins from, for example, the thick and thin filaments [99,100,101]. The expression of VCP/p97 is induced during sarcopenia, while the dominant-negative inhibition of VCP/p97 in the mouse muscle resulted in reduced fiber atrophy induced by starvation and denervation [99]. Overall, it is evident that, during sarcopenia or atrophy, muscle protein degradation is accelerated and appropriate proteasome activity is required to maintain physiological muscle function.

### 2.4. The Autophagy–Lysosome System (ALS)

**Figure 4:**
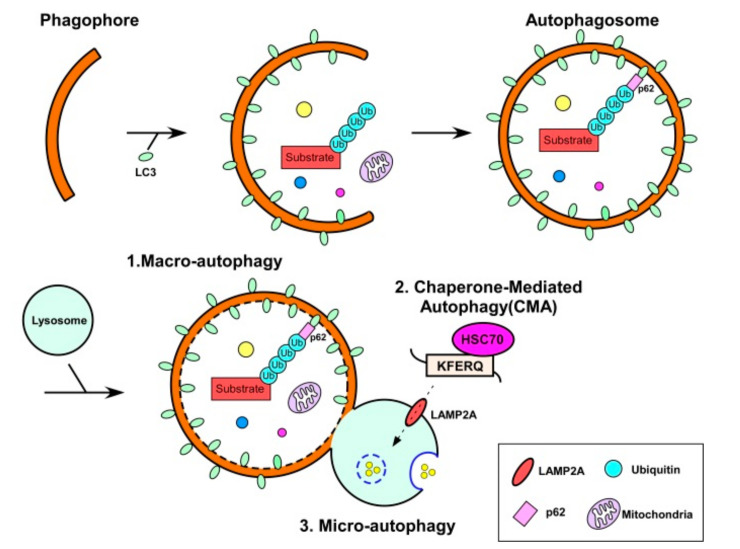
The autophagic degradation of proteins and organelles. Autophagy is the evolutionally conserved degradation pathway of intracellular components by the lysosomes. Autophagy involves initiation of the formation of the isolation membrane (phagophore), elongation of phagophore, maturation into autophagosome and fusion with lysosome for degradation. Three types of autophagy process have been described: macroautophagy (1), chaperone-mediated autophagy (CMA) (2), and microautophagy (3). The autophagosome biogenesis is executed by the sequential function of autophagy-related (Atg) proteins. Among Atg proteins, the formation and size of autophagosome are determined by two ubiquitin-like conjugation systems, the Atg12 and Atg8/LC3 systems. The carboxyl-terminal of LC3 is cleaved by the Atg4 to form LC3-I. The final LC3 lipidation with phosphatidylethanolamine (PE) to form LC3-II are orchestrated by Atg7, Atg3, and a complex consisting of Atg12-Atg5-Atg16L serving as E1, E2 and E3-like enzyme. The deficiency of basal autophagy may result in the accumulation of the abnormal aggregates of misfolded proteins in skeletal muscle during aging.

While the role of UPS in muscle atrophy has been extensively studied, only a few reports exist investigating that of ALS (Figure 4). The lysosome has been considered to be not involved in myofibrillar proteins [102]. The ALS is more energy demanding than the UPS, operating only at a basal level under normal conditions, but is induced when bioenergetic needs arise. It is possible that during the sarcopenic process, the overall changes to the cellular autophagic activity are similar to the effect on the proteasome. However, muscle-specific knockouts of autophagy-related genes result in atrophy and diverse myopathic phenotypes in mice [103,104]. The ALS appears to be involved in the homeostasis of skeletal muscle mass during the catabolic conditions, such as starvation, but is suppressed by the anabolic stimuli, such as protein intake and physical exercise [105,106,107]. Notably, many studies have reported that autophagy is induced by exercise in both mice and humans, suggesting the protective function of autophagy against the onset and progression of sarcopenia [39]. In aged muscles of mice and humans, autophagic adapter SQSTM1/p62 proteins accumulate with lipofuscin, an autoflorescent lipopigment formed with lipids, metals, and misfolded proteins, and this accumulation may suggest lysosomal dysfunction [108,109]. Moreover, the levels of several essential components of the autophagy machinery, such as Atg5, Atg7, and LC3, and autophagy-related modulators, such as Bcl-2, Bnip3, and autophagic-related GABA receptor associated protein 1 (GABARAP1), are decreased in aged rodent models [110,111], indicating that the overall activity of ALS is significantly impaired during aging. 

Mild induction or maintenance of autophagic flux appears to be critical for elimination of misfolded proteins and dysfunctional organelles; however, excessive induction of autophagy may aggravate skeletal muscle loss and, eventually, muscle cell death. The correlation between ALS and sarcopenia requires further comprehensive exploration. However, considering that the genetic manipulation to restore chaperone-mediated autophagy delays aging in mammals [110], it is possible that modulating ALS activity may be an effective strategy to maintain proteostasis in skeletal muscle. Importantly, the ALS substrates include not only proteins, but also lipids from the intracellular lipid droplets. A strong autophagic inhibition in Pompe disease, a lysosomal storage disorder manifesting as skeletal muscle myopathy [112], suggests that similar approaches may lead to beneficial consequences in sarcopenia as well. Furthermore, biogenesis of mitochondria declines with age [113]. Although a direct connection between mitochondrial quality control by autophagy (or mitophagy) and aging has not yet been determined, elevated levels of dysfunctional mitophagy likely contribute to the phenotypes and symptoms of age-related sarcopenia.

## 3. Therapeutic Potential of DHA in Sarcopenia by Modulating UPS and ALS

Currently, progressive resistance training with a high-protein diet is considered to be the most effective approach to improve muscle strength and function during age-related sarcopenia [114,115]. However, since most sarcopenic patients are sedentary and have only limited physical strength, there is a practical limitation in applying sufficient exercise regimen to treat sarcopenia. Moreover, the muscles of the older adults are often resistant to exercise-induced anabolic stimuli (referred to as anabolic resistance) [116]. Considering that there are no clinically proven drugs to treat sarcopenia, developing preventive and therapeutic strategies against sarcopenia is essential for healthy aging in the 21st century [34]. While the etiology of sarcopenia is not precisely understood, imbalanced protein metabolism in muscles is probably one of several direct causes. Therefore, a promising target for the treatment of sarcopenia is the suppression of UPS-mediated muscle protein degradation. Studies in different animal models have demonstrated that proteasome inhibitors may prevent muscle atrophy [117,118]; however, the UPS regulates a number of essential biological processes, and its strong suppression would interfere with critical cellular functions. Patients treated with bortezomib, an FDA-approved proteasome inhibitor for the treatment of multiple myeloma, display several side effects, including cardiac complications [119]. Therefore, this inhibitor might not be recommended as a treatment of sarcopenia due to higher risks among the older population. 

Growing evidence indicates that an intake of omega-3 PUFAs is beneficial to skeletal muscle not only by increasing skeletal muscle mass and strength, but also by improving recovery from pathologic muscle loss [15,120]. Omega-3 PUFAs are indispensable components of phospholipids that form the cell membrane structures [121]. Although there are several omega-3 fatty acids, alpha-linolenic acid (ALA; 18:3n-3), eicosapentaenoic acid (EPA; 20:5n-3), and docosahexaenoic acid (DHA; 22n:6n-3) are the most studied. ALA is present in plants and seeds and relatively abundant; its uptake is generally more than 15-fold greater than DHA and EPA [122]. DHA and EPA are both found in fish and in fish and krill oils [123]. DHA, in particular, is a primary structural component of the human brain, skin, sperm, and retina. Both DHA and EPA can be synthesized in the liver cells from ALA, but the conversion efficiency is only less than 15% and varies between individuals. For instance, Burdge et al. demonstrated that ALA is mainly converted to EPA and docosapentaenoic acid with no apparent conversion to DHA in young men [124]. Studies in cultured cells and animal models have shown robust evidence that omega-3 PUFAs may have anti-inflammatory properties and are generally known to lower blood pressure and to reduce the risk of cardiovascular diseases. The beneficial effect of omega-3 PUFAs on cardiovascular disease is still controversial since its effect was refuted by many clinical trials [125]. 

Omega-3 PUFAs in the plasma membranes are highly susceptible to peroxidation by the reactive oxygen species (ROS), which are continuously generated during mitochondrial metabolic reactions, and their production increases during the natural aging process [126,127]. Oxidized omega-3 PUFAs mediate the uncontrolled chemical reactions that form protein carbonyls, the direct markers of ROS-mediated protein oxidation and cellular oxidative stress [128,129]. It is notable that omega-3 PUFAs may dampen the inflammatory reactions, which is often persistently induced along with elevated levels of cytokines in the circulatory system with aging process (known as inflammaging). This inflammatory reaction may contribute to facilitated muscle protein degradation. Calorie restriction and its mimetics, such as NAD^+^ precursors, resveratrol, metformin, and rapamycin, appear to prevent inflammaging through modulating metabolic pathways including autophagy [130]. Induced autophagy via omega-3 PUFAs [129,131] may contribute to suppress inflammaging and delay the development of sarcopenia. 

Observational research and clinical trial data suggests that a higher omega-3 PUFA intake or higher plasma levels of omega-3 PUFAs may contribute to maintaining muscle mass and strength in older adults, preventing sarcopenic onset [14]. Multiple studies using C2C12 myoblasts indicate that EPA, but not DHA, effectively slow down muscle protein breakdown [15,132]. These outcomes probably originate from different concentrations of omega-3 PUFAs used because higher-dose supplement of DHA showed a similar effect on protein degradation as EPA [133]. To the best of our knowledge, the effects of EPA and DHA supplementation on muscle protein degradation rates in humans were never thoroughly analyzed yet [120]. Since EPA and DHA may serve as diverse downstream substrates and effectors of a number of cellular signaling pathways, it seems important to distinguish the underlying molecular mechanisms of EPA and DHA in vivo in the future studies. 

In addition to omega-3 PUFAs alone, which showed promising results as described above [14], when combined with resistance training, omega-3 PUFA supplements significantly augmented the benefits of exercise intervention [134,135,136]. The triple combination of exercise program and protein/omega-3 PUFA supplementation proved a mild but significant additive effect on muscle mass increase, compared to the exercise intervention alone [137]. Although the effects of omega-3 PUFAs varied in the combinatory treatment, probably due to the dose and duration of supplementation, these outcomes collectively suggest that omega-3 PUFA supplements, alone or combined with physical exercise, might have a beneficial effect to sarcopenic older adults.

A number of studies have established a positive effect of DHA and EPA intake on skeletal muscle homeostasis, although the exact mechanisms remain largely unknown. While supplementation with omega-3 PUFAs has been shown to have little effect on the basal rate of muscle protein synthesis, it effectively facilitates myogenesis in response to the anabolic stimuli [12,15]. A randomized controlled trial (RCT) revealed that omega-3 PUFA supplementation stimulates muscle protein synthesis and physical performance, measured by change in walking speed, in older adults [138,139,140]. Yoshino et al. conducted a comprehensive assessment of skeletal muscle gene expression profiles in the older population and found that omega-3 PUFA supplementation induces various signaling pathways involved in mitochondrial function and extracellular matrix organization, also reducing UPS-mediated proteolytic functions [141]. EPA, in particular, has been shown to decrease muscle protein degradation by preventing nuclear binding of the transcription factor nuclear factor κB (NF-κB), inducing MuRF1 expression [142]. Numerous studies have demonstrated that EPA alone effectively delayed muscle atrophy in different rodent models of muscle wasting, such as cachexia [143], acute starvation [144], hyperthermia [145] and sepsis [146], by suppressing UPS-mediated proteolysis.

**Figure 5:**
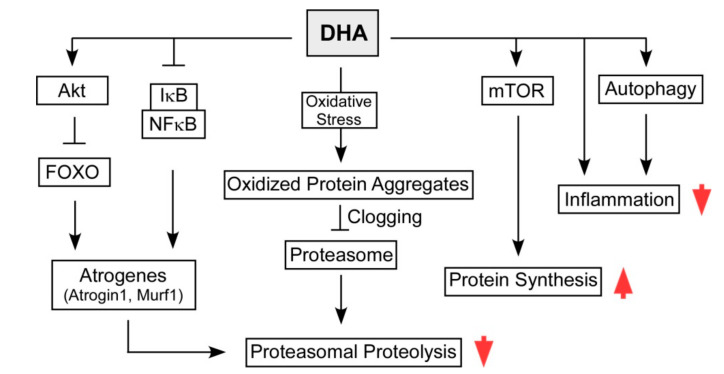
Overview of molecular mechanisms of docosahexaenoic acid (DHA) action potentially improving the skeletal muscle health and function through enhancing protein synthesis and reducing proteolysis and inflammation.

We have identified that DHA may delay muscle wasting through inhibition of the proteasome, providing a potential molecular mechanism of DHA-mediated sarcopenia therapy [16] (Figure 5). Treatment with DHA into either human cancer cells or human retinal pigment epithelium cells induced a significant amount of intracellular ROS comparable to menadione, a well-known ROS inducer. These increased ROS and resulting cellular oxidized proteins led to the accumulation of excess proteasome substrates (misfolded proteins or polyubiquitin conjugates), ultimately reducing proteasomal activity. The inhibitory effect of DHA on proteasomes is not due to direct inhibition of proteasome catalytic sites, but is rather a consequence of moderate levels of oxidative stress [16]. In fact, the DHA-induced oxidized proteins and proteasome inhibition are rescued by treatment with the antioxidant N-acetylcysteine, further supporting the hypothesis that DHA causes proteasomal dysfunction via generation of oxidative stress and subsequent proteasome overload. 

The inhibitory effect of DHA on proteasomal activity is also beneficial to the muscle cells under atrophic stress, since DHA can relieve dexamethasone (DEX)-induced muscle atrophy in C2C12 myotubes [16]. These effects were accompanied by recovery of MyoD, atrogin-1, and polyubiquitylated proteins, supporting the proposed mechanism of DHA protective effects during muscle wasting. Wang et al. and others have also observed that DHA attenuates protein degradation in C2C12 cells by regulating NFκB/PPARγ [147] and Akt/FOXO [148] pathways. Consistent with these, it has been shown in vivo that pre-feeding mice with DHA before fasting effectively prevented starvation-induced muscle atrophy by activating Akt- and AMPK-dependent signaling pathways and suppressing both UPS and ALS [149].

## 4. Conclusion and Future Perspectives 

The skeletal muscle accounts for approximately 40% of total body weight [150]. In addition to providing locomotive power, muscle also serves as a reserve of readily available peptides and proteins. Recent progress in sarcopenia research clearly indicates that muscle homeostasis is the result of a precise balance between the anabolic and catabolic processes. A small decrease in synthesis or increase in degradation, if sustained, can lead to a devastating pathological condition. The term sarcopenia was introduced nearly 20 years ago; now, with the worldwide population rapidly aging, this disease is currently gaining more considerable research interest and public attention. Although muscle mass and muscle strength are not always correlated, it is possible that the suppression of muscle protein breakdown is critical for the prevention and treatment of sarcopenia. Understanding detailed mechanisms involved in control of age-related changes in muscle proteins might offer a new therapeutic strategy for patients with sarcopenia. 

Omega-3 PUFA supplements might be an effective therapy to prevent or slow down sarcopenia. A number of studies based on rodents and humans have reported enhanced anabolic signaling in skeletal muscle [120]. Studies with C2C12 myotubes or fasted mice have demonstrated that treatment with EPA, but not DHA, significantly increased protein synthesis and decreased protein breakdown [132,144]. Many studies indicate EPA may effectively attenuate the UPS-mediated muscle protein degradation in cachexia murine models [143,144,151]. The mechanism of action mediating NF-κB inhibition appeared to be similar to the antitumor activity of bortezomib on multiple myeloma. Although the effects and mechanistic details of DHA on sarcopenia remain to be further elucidated, our current model suggests that DHA exerts its beneficial effects on muscle atrophy by decreasing proteasomal proteolytic activity by blocking it with oxidized proteins and excess proteasome substrates. However, the results regarding the effectiveness of DHA supplementation in attenuating muscle atrophy in humans are somewhat contradictory. In the RCTs performed by Smith et al., dietary supplementation with omega-3 fatty acids (containing 1.86 g EPA and 1.50 g DHA) significantly increased muscle protein synthesis delays the normal decline in muscle mass and function in older individuals [138,139] while a direct action of EPA and DHA on muscle protein synthesis or degradation was not investigated. Moreover, an 8-week administration of DHA preserved fasting (48 h)-induced muscle atrophy and proteolysis with upregulated autophagy [149]. The concentration, duration, and types of omega-3 PUFAs used in each experiment may directly affect the outcomes. Moreover, cells or organisms may have diverse and sometimes contradictory responses to DHA, which probably depend on the levels of oxidative stress induction. 

Growing evidence suggests that the mTOR signaling pathway influences longevity and aging. Inhibition of mTOR signaling with rapamycin (or its derivative rapalogs) is currently the only reliable pharmacological treatment option known to increase longevity in mice, as well as in yeast, worms, and flies, and to prevent age-related conditions in rodents, dogs, nonhuman primates, and humans [152]. mTOR complexes are serine/threonine kinases that lie downstream of Akt in the PI3 kinase pathway and regulate not only protein synthesis but also protein degradation through autophagy [21,153]. Under normal conditions, the free amino acids, the products of proteolysis, stimulate mTOR and facilitate protein synthesis through the downstream effectors, such as ribosomal protein S6 kinase 1 (S6K1) and eukaryotic translation initiation factor 4E binding protein 1 (4EBP1). As rapamycin, rapalogs, and rapamycin metabolites, both endogenous and dietary DHA can target mTOR, altering of downstream effector activation and subsequent protein synthesis [154,155]. However, this also upregulates cellular autophagy (contributing to the anti-inflammaging effects) and subsequently inhibits the UPS, which is more critical for muscle protein degradation. Therefore, the processes involving mTOR probably create a complex crosstalk between the pathways involved in protein synthesis and degradation, although the detailed mechanisms remain to be identified. Dual inhibitory effects of DHA on mTOR signaling and protein catabolism could be a potentially promising strategy to slow aging and extend a healthy lifespan. 

Currently, there is no pharmacological intervention method with a clear underlying molecular mechanism to prevent or treat sarcopenia. Considering the increasing recognition of individual and socioeconomic problems associated with sarcopenia, the potential of DHA as an anti-sarcopenic agent should be evaluated more thoroughly through global analysis of cellular oxidative stress and subsequent cellular proteome changes. Relatively newly instituted global standards for the screening and diagnosis of sarcopenia (International Classification of Disease, ICD-10-CM code.M62.84) can be applicable in both prospective and retrospective clinical trials [156]. A small anti-sarcopenic property of DHA would have a big impact on health and quality of life for the older population.
